# Factors affecting vection and motion sickness in a passive virtual reality driving simulation

**DOI:** 10.1038/s41598-024-80778-4

**Published:** 2024-12-04

**Authors:** Benjamin P. Hughes, Hassan N. Naeem, Nicolas Davidenko

**Affiliations:** Psychology Department, University of California, Santa Cruz, Santa Cruz, 95064 USA

**Keywords:** Motion perception, Virtual reality, Vection, Motion sickness, Driving simulator, Presence, Self-motion, Human behaviour, Psychology

## Abstract

The current study sought to examine factors that affect vection (the illusory experience of self-motion in the absence of real motion), visually-induced motion sickness, and one’s sense of presence in a passive virtual reality driving simulation by exposing participants to 60-s pre-recorded driving laps and recording their self-reported metrics as well as their head motion patterns during the laps. Faster virtual driving speed (average 120 mph vs. 60 mph) resulted in significantly higher ratings of vection and motion sickness. Reclined posture (30° back) was examined as a possible mitigating factor for sickness, but no significant effects were found. Expanding visual cues (representing forward self-motion) resulted in higher ratings of vection, motion sickness, and presence compared to contracting cues (representing reverse self-motion) and translational cues (representing lateral self-motion). When experiencing typical upright, world-aligned, forward-facing conditions, conformity to the median head motions along the yaw axis was associated with higher ratings of vection, motion sickness, and presence at slow speeds and with vection and presence at high speeds. These findings underscore the importance of head motion patterns as a metric for behavior and contribute to the general understanding of illusory self-motion perception.

## Introduction

The term “vection” is defined as the “illusory sense of self-motion^1^.” This phenomenon can occur when misleading visual signals convey a sense of motion, overriding the vestibular signals that would otherwise indicate a lack of movement. While vection-inducing circumstances are relatively rare in the natural world (e.g. looking out the window of a train as the adjacent train begins to move), virtual reality (VR) applications provide a host of scenarios in which vection can be experienced. VR head-mounted displays (HMDs) provide stereoscopic visual cues, which result in a stronger sense of vection than purely monocular cues^2^. By fully occluding real, stationary visual signals and replacing them with virtual visual signals that suggest self-motion, vection can be induced relatively quickly and easily using either simple stimuli such as optic flow starfields^3,4^ or more complex realistic virtual environments^5,6^.

While vection can be considered a desirable experience for some applications, some research has demonstrated its connection with the undesirable experience of visually-induced motion sickness (VIMS)^7^. In some contexts, this phenomenon is referred to as “cybersickness”^8^. Two major theories have been established to explain motion sickness, visually-induced or otherwise. Sensory conflict theory serves as the primary explanation for motion sickness, in which the sickness is thought to be triggered by a mismatch between the signals provided by two modalities (e.g. visual signals indicating forward motion and vestibular signals indicating no motion)^9–11^. Alternatively, postural instability theory posits that sickness results from an inability to adjust one’s posture in response to motion or perceived motion and the resulting sensorimotor conflict^12^. However, sensorimotor conflict cannot account for all instances of visually-induced motion sickness, as demonstrated by the induction of VIMS in scenarios where posture is fixed/stationary/restrained^13–16^. A review of vection and motion sickness studies has found that the relationship between vection and motion sickness is complex, with some studies finding a positive correlation between the phenomena and others finding no correlation^7^. This underscores the need to examine the various factors and circumstances that influence both experiences.

There is reason to believe that sex and exposure are important factors in motion sickness susceptibility. Some studies have found that women tend to experience a higher rate and severity of visually-induced motion sickness^17–22^, whereas other studies did not reproduce those findings and instead focused on individual differences in self-reported susceptibility as a more predictive factor^23,24^. Nevertheless, it is important to consider the potential for underlying physiological characteristics to drive motion sickness symptoms. Exposure and habituation to sickness-inducing stimuli have also been shown to influence the rate and severity of motion sickness. In the short term, VIMS increases over time with exposure^25^, but long-term habituation to such stimuli by way of repeated exposures (e.g. greater than 10 exposures) over the course of many days has been shown to reduce incidence of motion sickness in subsequent experiences^26^. This underscores the complex nature of the motion sickness mechanism as well as the importance of controlling for both individual characteristics such as sex and order effects in vection and motion sickness studies.

The influence of posture and position relative to gravity on vection warrants further study. There is evidence to suggest that changes in tilt and posture can affect the “weighting” of vestibular cues during the estimation of the subjective visual vertical (SVV), such that certain modalities are relied upon more heavily when their associated cues are more reliable^27^. When the reliability of visual cues is reduced, participants tend to rely more heavily on other modalities, such as haptic cues, when assessing ambiguous percepts like surface slant^28^. Numerous studies have also demonstrated that the perception of orientation is less accurate when the body (and therefore head) is recumbent (e.g. tilted 90 to 180°) compared to when it is upright^29^. Given these findings, it is reasonable to assume that changes in posture (e.g. sitting upright versus being reclined) could influence the subjective experiences of vection and/or motion sickness by way of sensory re-weighting of the vestibular signals.

Many vection studies use simple radial motion stimuli (e.g. white dots expanding radially from the center of an otherwise black display)^30–33^, but that is not the only motion stimulus that can result in the illusory experience of self-motion. Several studies have demonstrated the presence of vection in response to spiral and oscillating optic flow patterns^2,33^. Circular vection can be reliably induced by way of an optokinetic drum^34^, a large screen projection,^35^ or an equivalent presentation in virtual reality^36^. Along these lines, Keshavarz et al.^37^ found that the display-type of the vection stimuli (e.g. a single computer screen vs. a large dome projection screen) can have a strong impact on vection magnitude, onset time, and duration. With that in mind, very few studies have actively compared the magnitude of vection across different movement directions. Fujii and Seno^38^ examined vection responses across a variety of motion directions with two-dimensional motion stimuli, finding that direct movement (e.g. forward/backward and lateral movement) resulted in stronger vection responses than oblique movements. Pöhlmann et al.^39^ also manipulated movement direction in virtual reality, finding that forward/backward movement resulted in stronger vection than lateral movements.

Several studies have attempted to define objective behavioral and physiological correlates for vection^1^ as well as motion sickness^40^. Researchers have examined eye movements^31^, postural sway^41–43^, and EEG responses^7,44^ as possible correlates for vection, with mixed results. As Kooijman et al.^45^ points out, however, the majority of vection studies employ subjective measures due to the the fact that objective measures are often inaccessible to researchers or yet to be fully validated. Palmisano et al. (2015) notes that objective measures should always be paired with subjective self-reporting, seeing as there is no definitive physiological response that occurs only during vection^1^. Physiological measures for sickness, however, seem to be more reliable. Research by Keshavarz et al.^46^ has demonstrated that facial skin temperature and upper torso movement were both reliably correlated with higher rates of VIMS.

One potential behavioral marker for motion sickness that is still being explored is the rotational and translational movement of the head during sickness-inducing experiences. Several studies have employed trained neural networks that can reliably categorize the data from sick and non-sick participants based on a suite of physiological measures (heart rate, heart rate variability, breathing rate, and galvanic skin response)^47^, EEG data^48^, or in one case based only on their head and eye movements^49^. These findings point towards head movement as a consistent behavioral/physiological response to sickness-inducing visual stimuli that can be easily collected using the accelerometers present in all VR headsets. These studies represent an important step towards developing systems that could one day identify the early signs of VIMS and provide interventions to stop or mitigate sickness.

Previous studies have also examined head and eye motion as reliable behaviors during the experience of both real^50^ and simulated^51^ driving. For example, racing drivers reliably rotate their heads along the yaw and roll axes in a manner that is consistent with turns in the road^50^. Mestre and Authié^52^ suggest that these head movements are largely driven by visual cues rather than vestibular signals. Additionally, Li et al.^53^ examined the range and speed of head movement during a VR motion presentation and found that each had an effect on motion sickness. We posit that visually-induced vehicular motion simulation could also illicit head motion consistent with turns, and that head motion could be potentially be viewed as an implicit behavioral marker of perceived self-motion and/or sickness.

Another factor that has the potential for interaction with both vection and motion sickness is “presence,” which has been defined by Heeter^54^ as the subjective sense of “being there” in a virtual environment (as opposed to in one’s real environment) due to higher order factors like the physical realism and interactivity of the virtual environment. Slater et al.^55–57^ expanded on this definition and distinguished the term from immersion, which is driven by lower-order factors like the resolution of the screen and the degree to which non-virtual elements are shut out (e.g. by a head-mounted display and/or headphones). A review of studies investigating the connection between presence and cybersickness by Weech et al.^58^ found some evidence of a negative correlation between the two, such that those who felt more present in their virtual environments reported lower rates of sickness. However, other studies from the same review have discovered non-significant^59,60^ and even positive correlations^61,62^. The complicated relationship between these variables needs further investigation.

The current study intends to expand upon the literature concerning vection and motion sickness in virtual reality contexts by holistically examining self-reported experiences in combination with objective measures of head movement behavior. Participants experienced consistent presentation of vection-inducing stimuli using pre-recorded laps within a *passive virtual driving simulation*. In this context, *passive* is used to describe an implementation that does not present vestibular cues (e.g. via a motion platform) and does not allow for user control of the virtual vehicle (e.g. via a steering wheel controller). Participants experienced consistent presentation of vection-inducing stimuli using pre-recorded laps within a virtual reality head-mounted display, allowing for a repeated measures design that collected both head motion data during the laps and self-report measures (vection, motion sickness, and presence) following each lap. This study recorded head movement patterns over time as an implicit metric for behavior during VR experiences. By examining head movement over time across three axes, the current studies can investigate which patterns of motion tended to be associated with vection, sickness, and/or presence and whether those patterns represent normative or atypical movement. Investigating head movement behavior will allow us to examine whether a passive autonomous driving simulation elicits consistent head motion behavior in response to curves on the road and whether adherence to the normative pattern of movement is predictive of vection, presence, and/or motion sickness. In Experiment 1 (N = 52), driving speed, posture (sitting upright or reclined by 30°), and the alignment between the VR viewpoint and gravity (e.g. aligned or pitched upwards/downwards by 30°) were manipulated with the goal of discovering conditions in which posture and visual-vestibular misalignment could have a mitigating effect on sickness while retaining vection strength and presence. In Experiment 2 (N = 36), driving speed and driving direction (forward, reverse, or lateral) with the intent to examine whether forward self-motion is more compelling and sickness-inducing compared to reverse and lateral self-motion. We note that our manipulation of driving speed also manipulates optic flow (see Discussion for disambiguation).

## Results

### Experiment 1: posture, alignment, and speed

#### Sex and order

Participant sex and the order of conditions were initially considered as potential moderating between-subjects factors. Five-way ANOVAs were used to test the impact of these factors. The sex factor had 2 levels, and the order factor had 16 levels. Neither sex nor order had a significant effect on any of the self-reported variables. Sex did not have a significant effect on vection (*F*(1, 50) = 0.70, *p* = 0.41, $\eta ^2_p =0.03$), sickness (*F*(1, 50) = 1.14, *p* = 0.30, $\eta ^2_p = 0.04$), or presence (*F*(1, 50) = 0.93, *p* = 0.34, $\eta ^2_p = 0.04$). The order of conditions did not have a significant effect on vection (*F*(15, 25) = 1.63, *p* = 0.14, $\eta ^2_p = 0.49$), sickness (*F*(15, 25) = 0.82, *F* = 0.65, $\eta ^2_p = 0.33$), or presence (*F*(15, 25) = 1.16, *p* = 0.36, $\eta ^2_p = 0.41$). As such, these factors were not included in the following analyses. The following analyses utilized three-way ANOVAs that included three within-subjects factors (posture, alignment, and speed).

#### Vection

We assessed three measures of vection: magnitude (on a 1–20 scale), onset time (in seconds), and duration (in seconds). However, vection onset time and duration were only recorded for laps in which participants reported a 2 or greater on the vection magnitude scale (as 1 indicates a complete lack of vection). As such, we could only analyze vection onset time and duration data for 40 out of our 52 participants. The following analyses focus on vection magnitude, while the results for vection onset time and duration are available in the supplementary files.

##### Posture and alignment

**Fig. 1:**
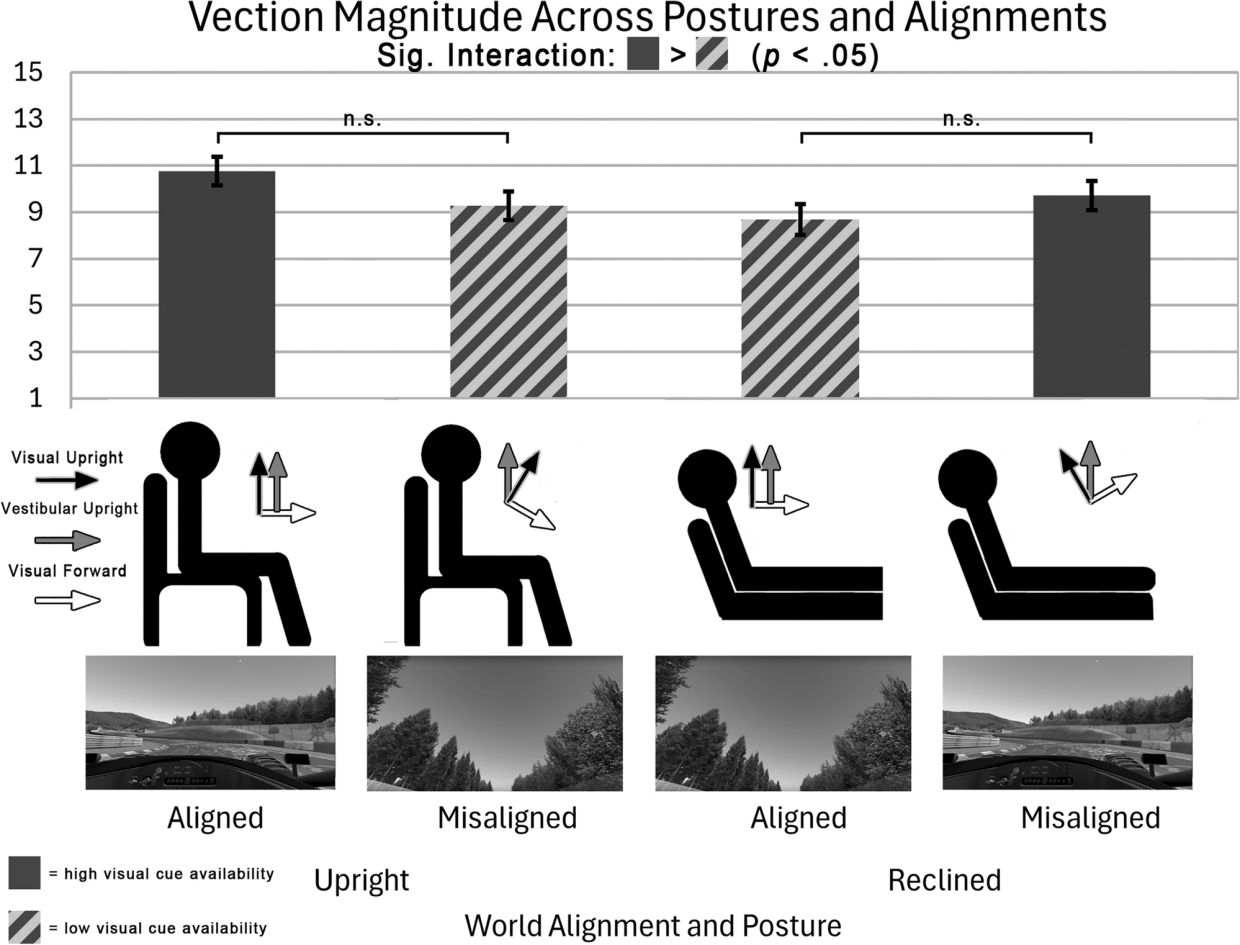
Bar graph showing the interaction between posture and alignment on vection magnitude ratings in Experiment 1. The diagrams and images below the bars represent the participants’ posture, alignment relative to gravity, and visual cues in each condition. The significant interaction between posture (upright vs. reclined) and world alignment (aligned vs. misaligned) shows that the conditions in which participants had a full view of the road ahead (solid grey bars) lead to stronger vection magnitude than the two conditions in which participants’ viewpoints were upward relative to the road (striped grey bars). “n.s.” indicates non-significant differences.

Although neither posture nor alignment had a significant main effect on vection magnitude (*F*(1, 51) = 3.87, *p* = 0.06, $\eta ^2_p = 0.07$; *F*(1, 51) = 0.38, *p* = 0.54, $\eta ^2_p = 0.01$), there was a significant interaction between posture and alignment, such that vection was more compelling when participants’ viewpoint was parallel to the virtual road (see Fig. 1 - upright/aligned and reclined/misaligned) compared to when participants’ viewpoint was pitched upward relative to the virtual road (see Fig. 1 - upright/misaligned and reclined/aligned) (*F*(1, 51) = 12.83, *p* < 0.01, $\eta ^2_p = 0.20$). This can be interpreted as a main effect of “visual cue availability,” such that vection was more compelling when visual cues to motion were more readily available (solid vs. striped bars in Fig. 1).

##### Speed

**Fig. 2:**
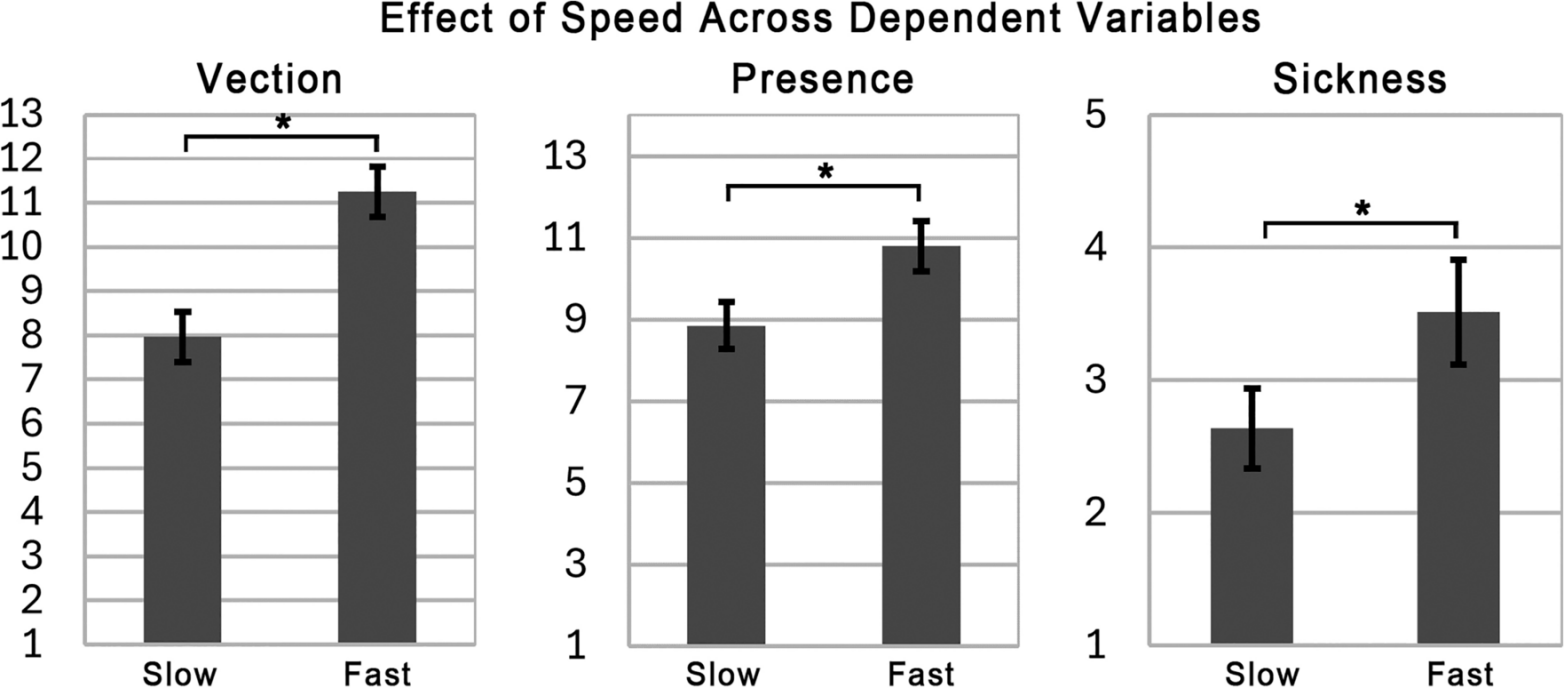
Bar graphs showing the effect of speed on vection, presence, and sickness ratings in Experiment 1. Asterisks indicate a significant difference (*p* < 0.05).

Speed had a significant effect on vection magnitude ratings, such that the higher speed condition resulted in a more compelling sense of vection (*F*(1, 51) = 71.98, *p* < 0.01, $\eta ^2_p = 0.59$). This result is shown in Fig. 2.

#### Sickness

##### Posture and alignment

Neither posture nor alignment had a significant main effect on sickness (*F*(1, 51) = 1.18, *p* = 0.28, $\eta ^2_p = 0.02$; *F*(1, 51) = 3.08, *p* = 0.09, $\eta ^2_p = 0.06$).

##### Speed

Speed had a significant effect on motion sickness ratings, such that high speed laps resulted in greater motion sickness severity (*F*(1, 51) = 11.28, *p* < 0.01, $\eta ^2_p = 0.18$). This result is shown in Fig. 2.

#### Presence

##### Posture and alignment

Posture had a significant effect on presence ratings, such that the upright condition resulted in a stronger sense of presence (*F*(1, 51) = 4.68, *p* < 0.05, $\eta ^2_p = 0.08$). Alignment had no significant effect on presence ratings (*F*(1, 51) = 1.17, *p* = 0.28, $\eta ^2_p = 0.02$).

##### Speed

Speed had a significant effect on presence ratings, such that the higher speed condition resulted in higher presence ratings (*F*(1, 51) = 40.91, *p* < 0.01, $\eta ^2_p = 0.45$). This result is shown in Fig. 2.

#### Correlations

Averaging vection and presence ratings across subjects and conditions, a Pearson’s *r* demonstrated that vection magnitude and presence had a strong positive correlation (*r*(50) = 0.89, *p* < 0.01). Motion sickness was not significantly correlated with vection or presence.

### Experiment 2: motion direction and speed

#### Sex and order

Participant sex and the order of conditions were initially considered as potential moderating between-subjects factors. Four-way ANOVAs were used to test the impact of these factors. The sex factor had 2 levels, and the order factor had 12 levels. Sex did not have a significant effect on vection (*F*(1, 15) = 0.07 *p* = 0.80, $\eta ^2_p = 0.01$), sickness (*F*(1, 15) = 1.42 *p* = 0.25, $\eta ^2_p = 0.09$), or presence (*F*(1, 15) = 0.23 *p* = 0.64, $\eta ^2_p = 0.02$). Order of conditions did not have a significant effect on vection (*F*(11, 15) = 1.18, *p* = 0.37, $\eta ^2_p = 0.47$), sickness (*F*(11, 15) = 0.81, *p* = 0.63, $\eta ^2_p = 0.37$), or presence (*F*(11, 15) = 0.90, *p* = 0.56, $\eta ^2_p = 0.40$). As such, these factors were not included in the following analyses. The following analyses utilized two-way ANOVAs that included two within-subjects factors (driving direction and speed).

#### Vection

As with Experiment 1, vection onset time and duration were only recorded for laps in which participants reported a 2 or greater on the vection magnitude scale (as 1 indicates a complete lack of vection). As such, we could only analyze vection onset time and duration data for 30 out of our 36 participants. The following analyses focus on vection magnitude, while the results for vection onset time and duration are available in the supplementary files.

##### Direction

**Fig. 3:**
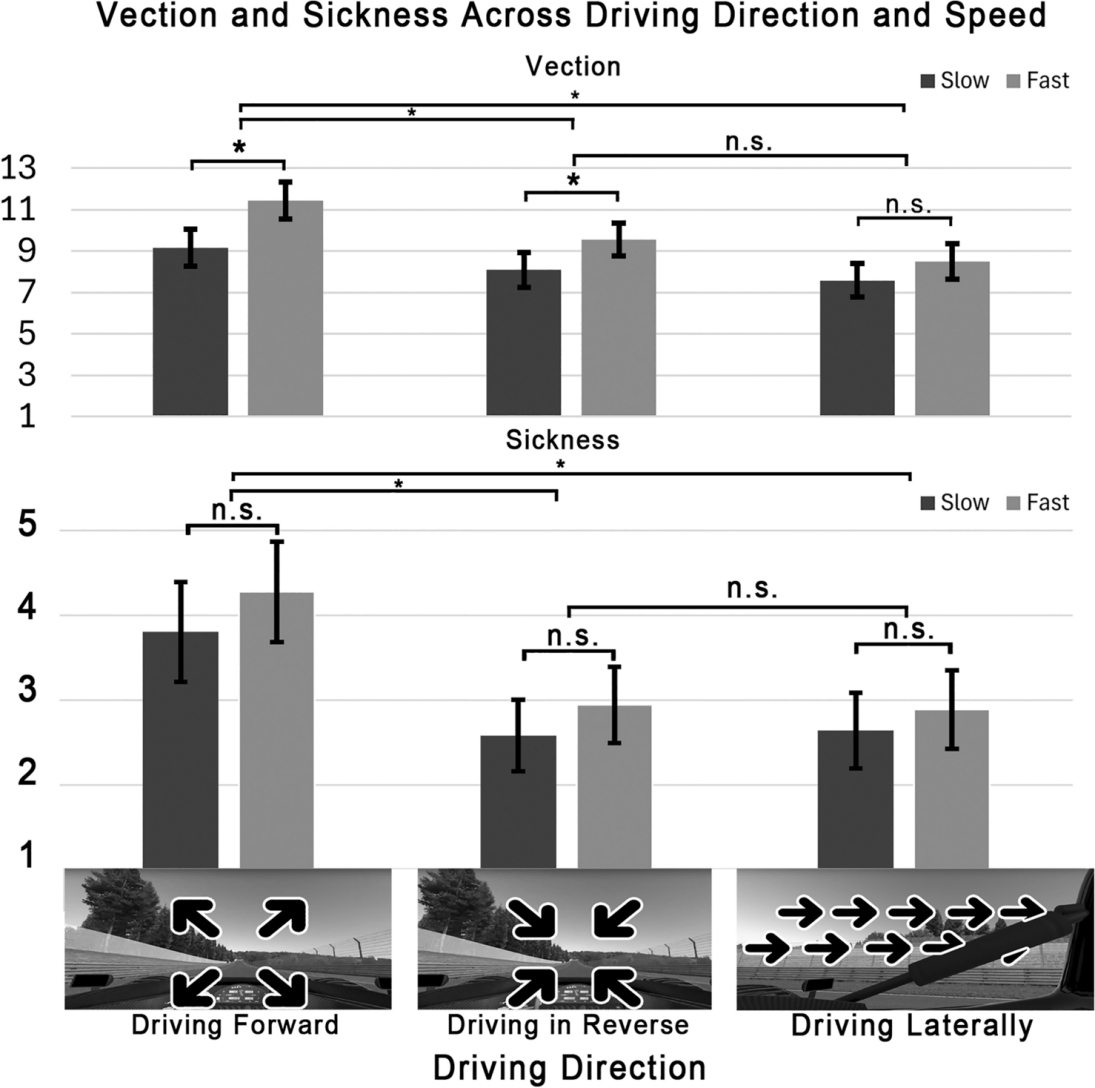
Bar graph showing the effects of speed and driving direction on vection magnitude (top) and sickness ratings (bottom) in Experiment 2. Asterisks indicate a significant difference (*p* < 0.05). “n.s.” indicates non-significant differences.

Driving direction had a significant effect on vection magnitude, such that driving forward resulted in a more compelling sense of vection than driving in reverse or laterally, and that driving in reverse resulted in a more compelling sense of vection than driving laterally (*F*(2, 70) = 5.05, *p* < 0.05, $\eta ^2_p = 0.13$). This result is shown in Fig. 3.

##### Speed

Speed had a significant effect on vection magnitude, such that the fast laps resulted in a more compelling sense of vection (*F*(1, 35) = 10.99, *p* < 0.01, $\eta ^2_p =0.24$). This result is shown in Fig. 3.

#### Sickness

##### Direction

Driving direction had a significant effect on motion sickness ratings, such that driving forward resulted in a greater motion sickness severity than driving in reverse or laterally (*F*(2, 70) = 6.65, *p* < 0.01, $\eta ^2_p = 0.16$). This result is shown in Fig. 3.

##### Speed

Speed had no significant effect on sickness (*F*(1, 35) = 1.72, *p* = 0.20, $\eta ^2_p = 0.05$).

#### Presence

##### Direction and speed

Neither driving direction nor speed had an effect on presence (*F*(2, 70) = 1.36, *p* = 0.26, $\eta ^2_p = 0.04$; *F*(1, 35) = 3.40, *p* = 0.07, $\eta ^2_p = 0.09$).

#### Correlations

Just as in Experiment 1, a Pearson’s r indicated that vection magnitude and presence ratings were positively correlated (*r*(34) = 0.92, *p* < 0.01). Sickness was not significantly correlated with vection or presence.

### Head motion analysis in the upright, forward-facing conditions combining data from experiments 1 and 2

We examined whether participants exhibited “normative” head motion behavior when experiencing a passive virtual driving simulation in typical conditions (e.g. upright, aligned, and facing forward). This condition was replicated exactly in both Experiments 1 and 2, which allows us to analyze head motion data in a larger sample (N = 88).

**Fig. 4:**
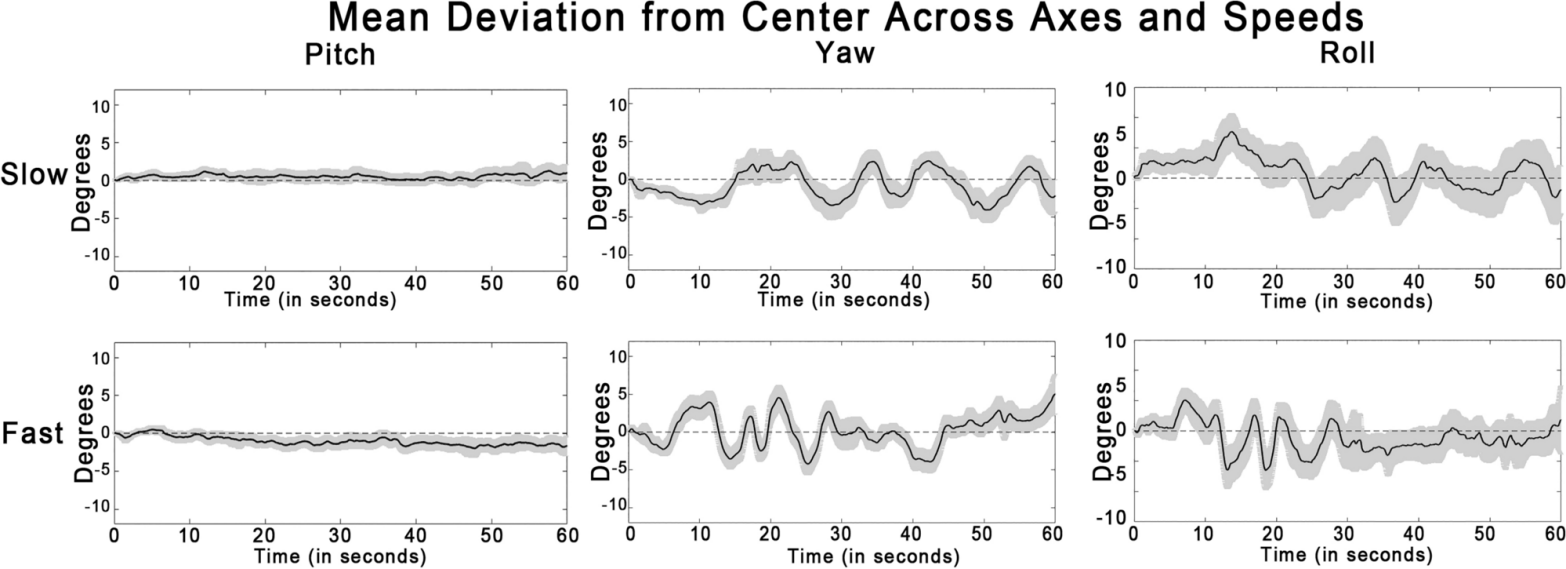
Line graphs plotting the mean deviation along each axis over time across speeds within the combined dataset. The black lines indicate the grand mean and the grey areas represent the standard error of that mean at each time point.

The data was recorded at 90 Hz (i.e., 90 datapoints per second) and was broken into three axes: pitch, yaw, and roll. The mean deviation from the center (the center being the participant’s default viewpoint, facing forward at the beginning of the lap) along each axis was analyzed and plotted over time. Figure 4 shows the mean deviation from the center over time along the pitch axis, yaw axis, and roll axis respectively.

#### Absolute head motion velocity

Overall head motion was quantified along each of the three axes (pitch, yaw, and roll) by computing the mean absolute velocity across each lap in degrees per second. The pre-recorded laps contain left and right turns thoughout, so we expected the absolute velocity of head motion along the yaw axis to be most pronounced. Indeed, absolute velocity of head motion along the yaw axis (*M* = 2.16, *M* = 2.61) was significantly greater than along the pitch (*M* = 1.17, *M* = 1.44) and roll (*M* = 0.9, *M* = 1.17) axes across both slow and fast laps respectively (all *p* s < 0.01).

#### Conformity of head motion

To analyze conformity of head motion to the normative pattern of head motion along each axis, the correlation between each participant’s head position and the median head position of every other participant was calculated across the 5400 time points in each lap (60 s at 90 Hz). Median head position was chosen over mean head position in order to minimize the influence of outliers.

The mean correlation coefficients across speeds and axes are shown in Table 1. Of note, along the yaw axis, the mean correlation to normative motion was 0.43 for slow laps and 0.47 for fast laps, indicating high conformity of head motion (*t*(87) = 16.10, *p* < 0.01; *t*(87) = 17.38, *p* < 0.01). Mean correlations for pitch and roll were significant, but lower compared to yaw (see Table 1). Paired *t*-tests confirmed that the average correlation between participants’ data and the median head motion was significantly greater than zero across all conditions and axes (also shown in Table 1). As expected, there was more conformity along the yaw axis than along the other two axes.

**Table 1:** Table containing values of one-sample *t*-tests of head motion across axes and speeds, along with the mean correlation coefficient for each speed and axis.

Axis	Driving speed	*t*-value (*p*)	Mean correlation to normative motion	*SD*
Pitch	Slow	**3.45 **(*p* < **0.01)**	0.07	0.20
	Fast	**9.86 **(*p* < **0.01)**	0.35	0.33
Yaw	Slow	**16.10 **(*p* < **0.01)**	0.43	0.25
	Fast	**17.38 **(*p* < **0.01)**	0.47	0.26
Roll	Slow	**10.61 **(*p* < **0.01)**	0.29	0.26
	Fast	**9.07 **(*p* < **0.01)**	0.29	0.30

Bold cells indicate significant *t*-tests (e.g. correlations to mean head movement significantly above zero).

Conformity to median movement along yaw axis was associated with higher rates of vection, sickness, and presence at slow speeds. At high speeds, conformity was associated with higher rates of vection and presence (but not sickness). In contrast, conformity to median movement along the pitch and roll axes were not significantly associated with any of the dependent variables (all *p* s > 0.14). The correlation coefficients and their associated *p*-values are displayed in Table 2.

Note that head turns along the yaw axis were closely aligned with the turns along the virtual track. The highest correlation between head motion data and car motion data (extracted from the simulator) occurred at a delay of approximately –750 ms, meaning that participants began making anticipatory head turns in the direction of an upcoming turn approximately 750 ms prior to the car beginning to turn.

**Table 2:** Table containing correlation coefficients between conformity to normative head motion across axes and dependent variables in the combined analysis.

Dependent variable	Driving speed	Head motion axes		
		Pitch	Yaw	Roll
Vection	Slow	0.01 (*p* = 0.91)	**0.33 **(*p* < **0.01)**	0.11 (*p* = 0.32)
	Fast	−0.03 (*p* = 0.81)	**0.29 **(*p* <**0.01)**	0.08 (*p* = 0.47)
Sickness	Slow	0.02 (*p* = 0.84)	**0.22 **(*p* < **0.05)**	0.10 (*p* = 0.36)
	Fast	−0.16 (*p* = 0.14)	−0.13 (*p* = 0.21)	0.09 (*p* = 0.43)
Presence	Slow	0.01 (*p* = 0.89)	**0.24 **(*p* <**0.05)**	0.01 (*p* = 0.89)
	Fast	−0.07 (*p* = 0.51)	**0.32 **(*p* <**0.01)**	0.11 (*p* = 0.30)

Bold cells indicate significant correlations.

## Discussion

The current experiments tested the effects of posture, alignment, driving direction, and speed on participants’ self-reported sense of vection, motion sickness, and presence. In Experiment 1, we found that higher speeds and more highly available visual motion cues result in a more compelling sense of vection. Higher speeds also increased both sickness and presence. In Experiment 2, we found that forward self-motion resulted in more compelling vection than reverse or lateral self-motion. Across both experiments, we found a very strong positive correlation between vection and presence ratings, but no correlation between sickness and vection or between sickness and presence. In addition, we developed a metric for examining behavior based on head motion velocity and position over time. In a combined analysis of head motion data across experiments, we found that conformity to normative motion along the yaw axis was correlated with vection, sickness, and presence, except in the fast conditions, in which conformity was not correlated with sickness. Further analysis of the combined head motion analysis revealed that movements along the yaw axis are anticipatory (as opposed to compensatory) relative to the perceived upcoming turns on the track.

Both experiments confirm that virtual driving speed plays a significant role in vection magnitude as well as motion sickness. While the present study only investigated two speeds, this opens the door for modeling vection ratings and sickness rates as a function of speed. Future studies could investigate whether the relationship between speed and vection/sickness is linear or nonlinear by presenting a wider variety of speeds. It should be noted that optic flow may play a confounding role in the effect of speed, such that the relationship between speed, sickness, and vection would not necessarily remain the same in response to a different stimulus. Follow-up studies could attempt to disambiguate the effects of outright speed (or perceived speed) from the effects of optic flow by replicating the experiment and manipulating the density of detailed objects and landmarks in the virtual environment and/or by systematically manipulating field-of-view.

Surprisingly, Experiment 1 did not show an effect of posture on any of the dependent variables. While this presents challenges for the idea of sensory re-weighting in the context of vection and sickness, there are several possible explanations for this null result. In this experiment, participants were only reclined by 30° from upright due to limitations on the driving simulation software. McManus et al.^63^ examined vection and visual reorientation illusions (VRIs) in upright and reclined positions, finding that those who were more susceptible to VRI experienced a stronger sense of vection and a greater emphasis on visual cues compared to vestibular cues. In that study, participants were fully reclined (e.g. parallel with the ground), which could indicate that sensory downweighting of the vestibular cues is sensitive to the degree of reclination and does not seem to take effect at only 30°. Future experiments could examine this by introducing a wider range of postural conditions that include full reclination.

The current studies observed an interaction between posture and alignment such that vection was more compelling in the conditions where visual cues were more available. When participants’ viewpoint was aligned with the camera orientation (regardless of their orientation relative to the real world), vection magnitude ratings were higher. Future studies of vection could investigate this further by testing the impact of visual cue availability across a wider range of positions and orientations relative to gravity.

Consistent with the findings of numerous vection studies^64–66^, both Experiments 1 and 2 showed a positive effect of speed on vection magnitude. However, a main effect of speed on sickness was observed only in Experiment 1. It is unclear why the effect of speed on sickness was not replicated in Experiment 2, especially given that the two studies shared an identical condition (the upright-aligned condition in Experiment 1 and the forward-facing condition in Experiment 2).

Experiment 2 demonstrated that forward-facing forward movement (expanding visual cues) resulted in stronger vection magnitude and higher sickness ratings compared to backward and lateral movement. In daily life, high-speed movement in reverse is extremely uncommon (as is lateral movement), which explains why the magnitude of the illusion along this axis is reduced. These findings are consistent with Bubka et al.^67^, which found that forward self-motion induced a greater vection magnitude than reverse and lateral movement and attributed that difference to the exposure-history-driven “neural expectancy” of forward motion. In other words, forward self-motion is more familiar and thus vection is easier to induce along that axis, resulting in a higher magnitude of vection, but also greater sensory conflict. Further investigation into the nature of illusory self-motion perception along multiple axes is warranted, especially considering its close relationship to VIMS.

When examining head movements during the upright-aligned conditions of both experiments, patterns of movement emerged, particularly along the yaw axis. Participants reliably turned their heads left and right by roughly 2–5° in response to the turns, which is consistent with numerous studies that have examined head movements during driving^50–52^. During the slow laps, conformity to the median yaw movement pattern was positively correlated with vection magnitude, sickness ratings, and presence ratings. However, during the fast laps, conformity was positively associated with vection and presence only. It is possible that normative head movement (in this case, shifting slightly with the turns) exacerbates the effects of motion sickness by maximizing the amount of visual signals that portray forward motion, but only when the stimulus is sufficiently slow. At fast speeds, the turns may be presented too quickly for an anticipatory head movement to make a difference in terms of sickness. Given the significant correlations between head motion conformity and the dependent variables (vection, sickness, and presence), further research into head motion behavior in other contexts is warranted. In particular, head movement patterns during self-directed movement should be examined in order to determine the impact of naturalistic deliberate head turning on self-motion and sickness experiences within virtual environments. Sickness classifiers using neural networks trained on head and eye movement data show remarkable promise^49^, but the underlying mechanisms that connect those movement patterns to sickness are still unknown and deserve further investigation. Our results show that head movements are highly consistent across participants, and that conformity to the average head motion pattern can serve as an implicit behavioral measure that tracks with subjective experiences of vection, motion sickness, and presence.

It should be noted that the observed head motions varied primarily along the yaw axis. This makes sense given that the visual stimulus rotated primarily along the yaw axis. It would be reasonable to assume that a similar pattern of movement conformity along other axes would arise in an alternate simulation that presents variable pitch and/or roll movement (e.g. a rollercoaster or flight simulation), but further research is necessary to determine if conformity to that potential pattern predicts vection and/or sickness.

When examined holistically, the results from these two experiments confirm several findings and open the door for future research within this field. This study has demonstrated that vection can be reliably induced using 60-s pre-recorded driving laps in virtual reality, and that speed plays a major role in the perception of self-motion. Future studies could expand upon this finding by investigating vection and sickness across a greater range of speeds. While previous vection studies have demonstrated the importance of speed^36^ and acceleration^64^, none have fully modeled vection as a function of speed/perceived speed. Such a study could potentially reveal the minimum and maximum speeds at which vection can be induced. This study also demonstrated that slightly-reclined posture has no significant effect on self-motion perception nor sickness. Follow-up studies should investigate the effect of posture across a wider range of positions ranging from fully-reclined to standing upright, allowing for the potential identification of ideal postures for the minimization of sickness and maximization of vection. These findings have implications for different seating and viewpoint positions in both VR driving simulators and real-life vehicles. While posture and viewpoint cannot be manipulated while driving, riding as a passenger or moving in VR allows for unconventional viewpoints (e.g. looking out the windows) and body postures that may have significant influences on rates of sickness and the experience of self-motion. Further investigation into the effects of these manipulations could potentially justify alternate seating positions and/or in-VR perspectives to enhance experiences of self-motion and mitigate motion sickness.

The results of this study should be interpreted with several potential limitations in mind. First and foremost, this study utilized a VR head-mounted display rather than a motion simulator platform. As such, the results can only be generalized to simulations that do not simulate vestibular/real motion cues. Second, this study involved passive presentation of self-motion rather than a self-directed, interactive experience. While this restriction allowed for a more controlled presentation of motion stimuli, it limits the ability to generalize this study’s findings to real-life driving scenarios. Another potential limitation of the current dataset is the lack of eye movement data. Given that previously established studies have demonstrated a functional relationship between eye and head movements during driving^68,69^, one can reasonably assume that participants were looking in the approximate direction that their head was pointing, but without having recorded this data, this cannot be confirmed. Finally, it should be noted that the method of presentation could have potentially influenced cognitive expectations of self-motion, given that existing studies have established the effects of contextual information, expectations, and plausibility on vection^70^ and motion sickness experiences^71^. Since there were only two speeds in the current study, participants were most likely able to differentiate a “fast” versus a “slow” lap and unconsciously fulfill the expectation of an appropriate sense of self-motion/sickness. Similarly, participants may have expected more or less vection/sickness in the unaligned conditions or in the conditions where visual cues were less available. Future studies could address this potential confound by implementing a between-subjects design (where participants are only exposed to one condition).

## Methods

All experiments were approved by the University of California, Santa Cruz institutional review board. All methods were performed in accordance with the relevant guidelines and regulations, including the Declaration of Helsinki. All participants gave informed consent to participate in the studies and were compensated using course credit.

### Experiment 1

#### Participants

4 participants that dropped out of the experiment early due to motion sickness were excluded from all analyses in order to preserve the counterbalancing of conditions within the experiment. The final dataset included 52 undergraduate students from the UCSC SONA subject pool (26 male, 26 female) between the ages of 18 and 26.

#### Design

This experiment employed a within-subjects, partially counterbalanced design. In this experiment, we manipulated body posture (upright or reclined by 30°), real/virtual world alignment (the virtual world is either aligned with the real world or pitched 30° down/upwards in the upright/reclined conditions), and the speed of the driving simulation (across all conditions). It should be noted that the virtual/real world alignment can also be expressed as a difference in available visual cues: in the real-world-aligned upright condition, visual cue availability is higher, whereas in the real-world-aligned reclined condition, visual cue availability is reduced. The perspective of the participants across the different cue availability conditions is shown in Fig. 1. In total, there were twelve trials across two main blocks, each containing two sub-blocks. In all conditions, the visuals followed live head tracking (i.e. participants could look freely around the virtual environment by turning their heads). The two main blocks manipulated body posture (upright or reclined, counterbalanced order). The sub-blocks manipulated the alignment of the real and virtual worlds. In “misaligned” conditions, a fixed pitch offset of +/– 30° was applied to the participants’ perspective. Within each sub-block, the stationary period was always experienced first, while the order of the following trials (full speed or half speed) was counterbalanced such that half the participants always experienced the half-speed lap first and half experienced the full-speed lap first. This fixed order of speed was chosen in order to avoid an excessive number of conditions to counterbalance.

#### Materials

The virtual reality stimuli for this experiment was presented on an HTC Vive head-mounted display. A computer tower with a GTX 970 Ti GPU and 16GB DDR3 RAM was used to run the associated software. The lap stimuli were presented through the “replay” feature of the popular consumer racing simulator iRacing. Several factors motivated the decision to use this program. The replay feature allows for consistent repeated presentations of a given lap or series of laps, such that each participant would experience the exact same driving lap. Furthermore, iRacing is popular in the racing simulation gaming community due to its visual realism, faithful reproduction of real-life tracks/cars, and customizability. An OpenVR plugin was utilized to allow for VR support. During the experiments, participants’ head rotation data was collected using Brekel OpenVR Recorder. This software converts live-action movements of the headset along three axes (yaw, pitch, and roll) to a .csv file. These files can then be analyzed to determine the participants’ head rotation velocity and deviation from center over the course of a given lap. The lap stimuli were recorded within iRacing. All laps took place within a virtual rendering of the Nurburgring Nordschleife race track. One lap was driven and recorded at an average speed of around 120 mph (top speed 140 mph). The slow version of this lap was constructed by presenting the original lap at half-speed (resulting in an average speed of around 60 mph, top speed 70 mph). Each lap lasted exactly 60 s and featured no audio. It should be noted that the slow version of the lap covers half the distance of the fast version. We felt that it was more important to keep the timing (rather than the distance traveled) consistent across speed conditions, especially when recording onset time and duration of vection experiences.

#### Procedure

Participants arrived, signed consent forms, then put on the head-mounted display with the help of a research assistant. They were then randomly assigned to a seating position (upright or reclined) which they maintained for the first main block of six trials, before switching to the other seating position. Within these main blocks, they were randomly assigned to a sub-block of three trials. Each block of six trials was broken into two sub-blocks of three trials each. Based on pilot experiments, we expected most participants to feel a compelling sense of vection and presence in the simulation and that sickness would be relatively low. We also found that participants tended to get confused when the rating scale questions differed in scale. Ultimately, we chose the 20-point, one-item Fast Motion Sickness scale (FMS)^72^ to assess sickness because of its reliability and brevity. While various measures to assess vection have been employed in other studies (e.g. binary response or alternative forced choice)^45^, we chose a one-item, 20-point scale to measure vection magnitude in order to remain consistent with the sickness scale and avoid participant confusion. Similarly, we chose a single-item, 20-point scale to assess presence. An advantage to using rating scales to assess the quality of the vection experience is that they allow for the detection of potentially small differences across experimental conditions^45^. Before any trials began, participants were briefed on the questions they would be asked after each trial. The vection magnitude question was phrased as follows, “How compelling was your sense of self-motion during the trial, on a scale of 1–20? 1 means you didn’t feel any self-motion at all, and 20 means you completely felt as if you were moving.” Sickness and presence ratings were phrased similarly, with the sickness end-points being 1 (“no sickness at all”) and 20 (“completely sick”) and the presence end-points being 1 (“I completely felt as if I was sitting on a chair/bed here in the lab”) and 20 (“I completely felt as if I was sitting in a race car on the track”). Participants were also instructed that each trial would last 60 s, and that they would be asked to report at what point in time they began to feel like they were moving and for how long (if at all). The first trial of each sub-block was stationary, to establish a baseline sense of motion sickness and presence. The next two trials were presented in a counterbalanced order of the laps described in the Stimuli section (full speed, half speed). After each trial, the participant was asked to self-report vection magnitude (on a 1–20 scale), vection onset time (in seconds), vection duration (in seconds), motion sickness (1–20 scale), and presence (1–20 scale). Following the completion of the first main block, participants completed a midpoint survey assessing more detailed measures of vection, motion sickness, and presence. Then they completed the second main block in the alternate seating position (upright or reclined). Following the second block, the participants completed the same survey again followed by a demographic survey. Throughout all trials, rotational and positional data was recorded continuously.

### Experiment 2

#### Participants

3 participants that dropped out of the experiment early due to motion sickness were excluded from all analyses in order to preserve the counterbalancing of conditions within the experiment. The final dataset included 36 undergraduate students from the UCSC SONA subject pool (15 male, 21 female) between the ages of 18 and 41.

#### Design

This experiment employed a within-subjects, partially counterbalanced design similar to Experiments 1. In this experiment, we manipulated the driving direction the participant was exposed to (driving forward, in reverse, or laterally), and the speed of the driving simulation (across all conditions). In the forward and reverse conditions, the participant is facing out the front of the virtual car, but the direction of the car’s movement is manipulated (e.g. driving forward, resulting in expanding visual motion cues, and driving in reverse, resulting in contracting visual motion cues). In the lateral condition, the participant is turned 90° to the right as the car moves forward. Every participant experienced all three driving direction blocks in a counterbalanced order. Speed order was kept consistent within each participant (e.g. slow laps first in each block or fast laps first in each block) and counterbalanced across conditions. The three main blocks manipulated driving direction in a counterbalanced order. Within each block, the stationary period was always experienced first, while the order of the following trials (full speed or half speed) was counterbalanced such that half the participants always experienced the half speed lap first and half experienced the full-speed lap first. This fixed order of speed was chosen in order to avoid an excessive number of conditions to counterbalance.

#### Materials

Experiment 2 utilized the same software, hardware, and stimuli (e.g. lap recordings) as Experiment 1. The different driving direction conditions were manipulated as follows: the expanding cue conditions were functionally the same as the upright-aligned conditions in Experiment 1, the contracting cue conditions consisted of the same laps played in reverse, and the translational cue conditions involved rotating the in-game camera by 90° along the yaw axis (then playing the standard forward-driving laps while the participant faced out the right side of the car).

#### Procedure

Just as in Experiment 1, participants arrived, signed consent forms, then put on the head-mounted display with the help of a research assistant. They were then assigned to one of three driving direction conditions (forward, in reverse, or lateral) which they maintained for the first block of three trials. The first trial of each block was always stationary, to establish a baseline sense of motion sickness and presence. The next two trials were presented in a counterbalanced order of the laps referenced in the Stimuli section (full speed, half speed). During all trials, participants were instructed to look forward (relative to the real world, not the virtual world), but their head was not physically constrained in any way. After each trial, the participant was asked to self-report vection magnitude (on a 1–20 scale), vection onset time (in seconds), vection duration (in seconds), motion sickness (1–20 scale), and presence (1–20 scale). The phrasing of the instructions and rating scales was the same as Experiment 1. Following the completion of all three blocks, participants completed a survey assessing more detailed measures of vection, motion sickness, and presence, as well as demographic information.

## Data Availability

The datasets and corresponding code files generated and analysed during the current study are available from the corresponding author on reasonable request.
